# Bayesian Calibration to Address the Challenge of Antimicrobial Resistance: A Review

**DOI:** 10.1109/ACCESS.2024.3427410

**Published:** 2024

**Authors:** Conor Rosato, Peter L. Green, John Harris, Simon Maskell, William Hope, Alessandro Gerada, Alex Howard

**Affiliations:** 1 Department of Pharmacology and Therapeutics, University of Liverpool, L69 7BE Liverpool, U.K.; 2 Department of Mechanical Engineering, University of Liverpool, L69 7BE Liverpool, U.K.; 3 United Kingdom Health Security Agency (UKHSA), SW1P 3JR London, U.K.; 4 Department of Electrical Engineering and Electronics, University of Liverpool, L69 7BE Liverpool, U.K.

**Keywords:** Antimicrobial resistance, antimicrobial stewardship, approximate Bayesian computation, Bayesian inference, epidemiology, Markov chain Monte Carlo, sequential Monte Carlo

## Abstract

Antimicrobial resistance (AMR) emerges when disease-causing microorganisms develop the ability to withstand the effects of antimicrobial therapy. This phenomenon is often fueled by the human-to-human transmission of pathogens and the overuse of antibiotics. Over the past 50 years, increased computational power has facilitated the application of Bayesian inference algorithms. In this comprehensive review, the basic theory of Markov Chain Monte Carlo (MCMC) and Sequential Monte Carlo (SMC) methods are explained. These inference algorithms are instrumental in calibrating complex statistical models to the vast amounts of AMR-related data. Popular statistical models include hierarchical and mixture models as well as discrete and stochastic epidemiological compartmental and agent based models. Studies encompassed multi-drug resistance, economic implications of vaccines, and modeling AMR in vitro as well as within specific populations. We describe how combining these topics in a coherent framework can result in an effective antimicrobial stewardship. We also outline recent advancements in the methodology of Bayesian inference algorithms and provide insights into their prospective applicability for modeling AMR in the future.

## I Introduction

The routine application of Bayesian algorithms, including Markov Chain Monte Carlo (MCMC) and Sequential Monte Carlo (SMC) methods, in the field of health sciences has been instrumental in combining disparate data sources to make disease-related inferences when closed-form solutions are not known. An advantageous feature of using Bayesian methods is the incorporation of prior knowledge through probability distributions associated with each data source, distinguishing Bayesian methods from other prediction-based methods. Calibrating models using Bayesian methods allows the propagation of uncertainty through the model, providing probability distributions over predictions. Despite the utility of Bayesian methods, a potential obstacle to widespread usage is the required computational demand, which can be addressed by deploying Bayesian algorithms across multiple computers in a distributed framework. As healthcare-related data continues to grow in both magnitude and complexity, the computational resources required for gaining insights into challenges like AMR also increase. A number of review articles outlining the theory, principles, challenges and working examples of performing Bayesian analysis in healthcare-related disciplines can be found in [1], [2], and [3].

The World Health Organization (WHO) has recently classified AMR as a significant global threat to both health and society at large [4]. While the discovery of new antimicrobial agents remains crucial in combating AMR, the effectiveness of this strategy is limited without a comprehensive understanding and optimization of both existing agents and future agents yet to be licensed. Certain challenges particular to AMR are especially suited to modelling using Bayesian techniques. For example, imperfect diagnostic criteria and laboratory tests can lead to uncertainty in the underlying patient disease and colonisation state. Furthermore, the impact of interventions extends beyond the duration of most clinical studies, since the generation of AMR in a population can take years to manifest.

Antimicrobial Stewardship (AMS) constitutes a comprehensive initiative aimed at fostering the judicious utilization of antimicrobials based on data obtained from the delivery of routine clinical care [5]. The primary goals of AMS include reducing AMR and curbing the dissemination of infections stemming from multidrug-resistant organisms, all while taking into account the individual’s infection risk [6]. Effective AMS relies on a combination of measures, including infection control, precise detection of AMR in diagnostic laboratory settings and population-level surveillance. Quantifying these measures in isolation is challenging for several reasons, including insufficient quantities of data, unmonitored resistance rates, the complexity of required models, and the dynamics of co-infection with sensitive and resistant strains [7]. The Bayesian analyses conducted in the reviewed articles have the potential to bridge the gap between Artificial Intelligence (AI) and AMR, as outlined in [8].

The contribution of this paper is to provide a comprehensive and up-to-date overview of the current state of knowledge in the combined topics of AMR and Bayesian modelling. Some of the key points are summarised below:

- **Integration of Bayesian modelling in AMR research**: This review aims to bridge the gap between AMR and Bayesian modeling by exploring and reviewing studies and applications where Bayesian methods have been employed to analyse and model AMR data. Several review articles have detailed the applications of Machine Learning (ML) and AI in tackling antimicrobial resistance (AMR) [9], [10], [11], [12]. However, this review article is the first to exclusively focus on Bayesian methods.
- **Uncertainty Quantification**: This paper highlights the importance of Bayesian modelling in quantifying uncertainty. Bayesian methods allow for the incorporation of prior knowledge, which is especially crucial when dealing with incomplete or heterogeneous data sources.
- **Prediction and Forecasting**: This paper outlines how Bayesian modelling is an effective method for predicting and forecasting trends in AMR. Selected studies have shown how the evolution of resistance patterns within certain populations are affected by interventions over time.
- **Methodological Overview**: A detailed methodological overview of the development and progression of Bayesian inference algorithms is provided, with a specific emphasis on efficiently deploying such models on distributed computing platforms. A description of commonly used models, such as hierarchical models, and their application in understanding the dynamics of AMR is provided.
- **Identifying Gaps and Challenges**: Finally, this paper identifies gaps in the current literature and the challenges associated with the application of Bayesian modelling in AMR research.

The structure of this review is organised as follows: Section IV defines Bayes’ theorem, while Sections IV-A and IV-E outline MCMC and SMC methods, respectively. Section V covers statistical methods for evaluating Bayesian algorithms. In Sections VI, a comprehensive list of techniques to model AMR is provided. Finally, Section VIII discusses potential directions for future work, including recent advancements in both modeling and sampling algorithms.

### A Search Criteria

This review encompasses studies examining a diverse array of causative pathogens, as identified in internationally-recognised lists which highlight their critical importance in AMR. These lists include the WHO Priority Pathogens List and the ESKAPE pathogens [55], [56]. The search criteria for this review did not identify Bayesian modelling studies of AMR in *Helicobacter pylori, Campylobacter* spp and *Enterobacter* spp, which are pathogens identified in the prior lists. As outlined in Table 1, the most commonly studied pathogens are *Mycobacterium tuberculosis* and methicillin-resistant *Staphylococcus aureus* (MRSA), highlighting their significant impact on health outcomes. For instance, *M. tuberculosis* can develop resistance to treatment and, due to its mode of transmission, can lead to community outbreaks of multidrug-resistant *M. tuberculosis*. Similarly, MRSA can have severe consequences within healthcare settings. Since both of these pathogens are communicable, Bayesian calibration is a widely used approach for epidemiological transmission models.

The search engines considered for the advanced search of this review included Web of Science, PubMed-MEDLINE and Scopus. The search was not restricted by date and after publications with no full text available, review articles with no novelty and duplicates were eliminated, a total of 567 articles were considered. To be considered, an article had to include Bayesian modelling of bacterial AMR in humans (veterinary and environmental studies were excluded). Viral and vector-borne diseases such as HIV and malaria were not considered. Population pharmacokinetic (popPK) models and phylogenetic tree analyses were considered out of scope and excluded. However, a brief introduction to popPK and phylogenetic trees are provided in Section VIII-D. In terms of Bayesian modelling, studies had to use Approximate Bayesian Computation (ABC), MCMC or SMC methods. Therefore studies that employed, for example nïve Bayes ML classifiers and Bayesian information criterion, were not included. This resulted in 43 studies. Additionally, references of selected papers and Google Scholar were manually searched and this identified 22 research articles that were not captured by the advanced search queries.

## II Contextual Example

Let’s consider the scenario where we aim to estimate the prevalence of AMR, characterized by parameters of the model that has *D* dimensions, ***θ*** ={*θ*_1_, *θ*_2_, …, *θ*_*D*_}, within a population using observed laboratory test outcomes denoted by **y** = {y_1_, y_2_, …, y_*N*_}. Each element in **y** signifies whether the bacterium in the sample exhibits resistance (y = 1) or susceptibility (y = 0) to the antibiotic being studied.

## III Bayes’ Theorem

Bayes’ theorem, outlined in (1), is a mathematical formula used to determine the probability of ***θ*** conditional on **y**:

(1) $$p(\theta |\text{y})=\frac{p(\text{y}|\theta )p(\theta )}{p(\text{y})},$$

where *p*(**y|*θ***) is the probability of **y** given ***θ*** and *p*(***θ***) and *p*(**y**) are the independent probabilities of ***θ*** and **y**, respectively. Equation (1) provides a method for revising predictions after considering newly obtained evidence.

## IV Bayesian Inference

Bayesian inference uses Bayes’ theorem to update ones personal belief after observing the data. The parameters of a statistical model are often unknown and difficult to measure directly and need to be inferred from the data. Prior information can be attributed to ***θ*** in the form of a probability distribution, *p*(***θ***). This prior distribution may encapsulate our initial beliefs about the prevalence of AMR within the population, representing uncertainty regarding ***θ*** before encountering any data. The likelihood is given by the conditional probability of **y** given the prevalence of resistance, *p*(**y**|***θ***). The likelihood describes the probability that the data was produced from the statistical model whose parameters, ***θ***, are fixed to a particular value. If the prior and likelihood can be defined, the parameter posterior distribution, *p*(***θ***|**y**), can be calculated using Bayes’ theorem:

(2) $$p(\theta |\text{y})=\frac{p(\text{y}|\theta )p(\theta )}{p(\text{y})}=\frac{p(\text{y}|\theta )p(\theta )}{\int_{\theta }p(\text{y}|\theta )p(\theta )d\theta }\propto p(\text{y}|\theta )p(\theta ).$$

Bayes’ theorem expresses a probability distribution over ***θ*** which is conditional on **y** and allows us to update our belief about the prevalence of resistance based on the observed test results.

If the likelihood and prior can be easily defined, the calculations are often algebraic or can be performed using statistical software. This explicitness allows for easy computation of the posterior distribution. When a closed-form solution for the integral in equation (2) cannot be found, approximating it can pose challenges, particularly in high dimensions where it may become exceedingly difficult or even impossible. In more complex scenarios, where analytical solutions are not feasible, numerical methods like MCMC may be employed to draw samples from the posterior. The posterior is typically estimated up to a normalisation constant, given by the integral which marginalises out ***θ***.

Table 2 outlines a number of Bayesian inference algorithms as well as corresponding research studies in which they have been applied when modelling AMR. The algorithms include MCMC, ABC and SMC based algorithms which are described in Sections IV-A, IV-C and IV-E, respectively.

### A MArkov Chain Monte Carlo

MCMC methods provide a mechanism for sampling from an arbitrary probability distribution *π*(***θ***) that, in the context of Bayesian inference, is set proportional to the posterior:

(3) π(θ)∝p(y∣θ)p(θ).

MCMC methods generate a Markov chain that forms a sequence of correlated samples from *π*(***θ***). A Markov chain is Markovian in that the current sample only depends on the previous. The transition operator *T* (***θ***, ***θ***′) describes the probability of going from the current sample ***θ*** to the proposed sample ***θ***′. For a Markov chain to generate samples from *π*(***θ***), it must meet specific criteria. These criteria encompass the chain being ergodic and possessing a stationary distribution. The stationary distribution is the distribution that the Markov chain converges to after running for a sufficiently long time. It represents the desired distribution from which we want to sample.

Detailed balance is a condition that the transition probabilities of the Markov chain must satisfy to ensure that the stationary distribution is indeed the desired target distribution. Mathematically, detailed balance is expressed as:

(4) $$\pi (\theta )T\left(\theta ,\theta^{\prime }\right)=\pi \left(\theta^{\prime }\right)T\left(\theta^{\prime },\theta \right).$$

The time until the Markov chain has converged and reached its stationary distribution is dependent on its initial starting point in the parameter space and choice of transition operator *T* (***θ***, ***θ***′). Popular MCMC methods are described in the subsequent sections.

### 1) Metropolis-Hastings Random Walk

As seen in Table 2, the Metropolis-Hastings Random Walk (MHRW) is the most commonly used MCMC algorithm when modelling AMR. It splits *T* (***θ***, ***θ***′) into two distinct steps: the proposal step and accept/reject step.

The proposal step draws a vector of states ***θ***′ from a proposal distribution *q*(***θ***′|***θ***). A simple example of a MHRW proposal is the Normal distribution:

(5) $$q\left(\theta^{\prime }|\theta \right)=N\left(\theta^{\prime };\theta ,\text{Σ}\right),$$

where **Σ** ∈ *R*^*D*×*D*^ is the covariance, which is selected by the user.

The accept/reject step ensures that the Markov chain explores the state space efficiently by probabilistically accepting proposed moves, while detailed balance ensures that the stationary distribution of the Markov chain is the desired target distribution. Together, these components ensure the correctness and convergence of the Metropolis-Hastings algorithm. The acceptance probability, *α*(***θ***, ***θ***′), is given by

(6) $$\alpha \left(\theta ,\theta^{\prime }\right)=\text{min}\left\{1,\frac{\pi \left(\theta^{\prime }\right)q\left(\theta |\theta^{\prime }\right)}{\pi (\theta )q\left(\theta^{\prime }|\theta \right)}\right\}.$$

If the proposal is symmetric *q*(***θ*** |***θ***′) = *q*(***θ***′|***θ***), as is with the MHRW, the acceptance probability simplifies to

(7) $$\alpha \left(\theta ,\theta^{\prime }\right)=\text{min}\left\{1,\frac{\pi \left(\theta^{\prime }\right)}{\pi (\theta )}\right\}.$$

The acceptance rate is a measure of the proportion of proposed moves that are accepted during the sampling process. Proposing steps that are too small can result in slow exploration which can lead to ***θ***′ being proposed too close to ***θ***, causing high autocorrelation within the Markov chain. Taking steps that are too large can result in low acceptance rates and inefficient sampling as ***θ***′ may fall outside high probability regions of *π*(***θ***).

**Figure 1:**
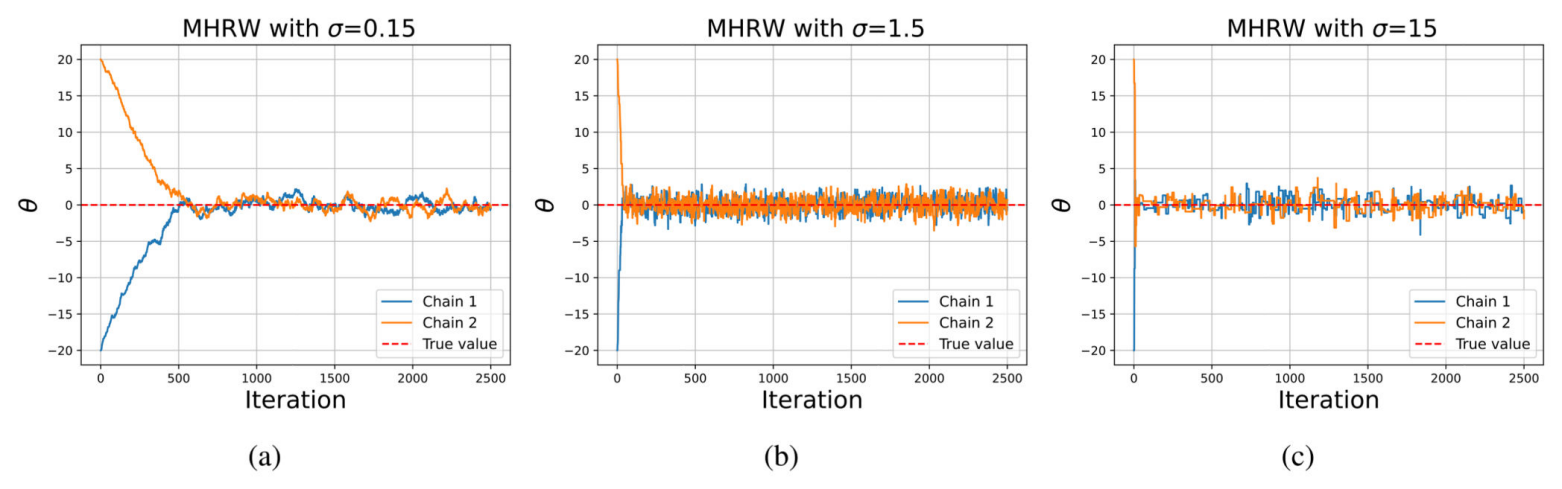
Two Markov chains with different initial starting points when sampling from a 𝒩(0, 1) when using (a) *σ* = 0.15, (b) *σ* = 1.5 and *σ* = 15 in the proposal distribution. The horizontal dashed red line is the true value. The average acceptance rate for both chains in subplots (a), (b) and (c) are 0.906, 0.602 and 0.084, respectively.

Figure 1 illustrates the importance of selecting an appropriate *σ*. It is evident that using a smaller value of *σ* in Figure 1(a) takes longer to reach the probability mass when compared to using a larger value in Figure 1(c). The acceptance rate for Figures 1(a), (b) and (c) are 0.906, 0.602 and 0.084, respectively. To implement the acceptance step in (6) algorithmically, a random variable *u* is drawn from a Uniform distribution on [0, 1]. If *u* < *α*(***θ***, ***θ***′) then ***θ***′ is accepted as the next state of the Markov chain. If *u* > *α*(***θ***, ***θ***′), ***θ***′ is rejected and the Markov chain’s state remains equal to ***θ***. This process is repeated for *M* MCMC iterations.

The steps performed by the MHRW algorithm are outlined below:

1) Assign and draw initial values for ***θ*** from respective prior distributions.
2) Following (3), calculate the parameter posterior distribution by multiplying the prior and the likelihood.
3) Propose a new vector of ***θ***′ via the proposal distribution in (5).
4) Accept ***θ***′ using the acceptance step in (6).
5) Repeat steps 2-4 for *M* iterations to produce a Markov chain consisting of *M* samples.
6) Perform diagnostic checks (see Section V) on the resulting Markov chain to determine if the sampling process is producing samples that are representative of *π*(***θ***).

The MHRW sampler may encounter the “curse of dimensionality”; as the dimensionality of ***θ*** increases, efficiently sampling from *π*(***θ***) becomes increasingly challenging.

### 2) GIBBS

Similarly to the MHRW sampler, Gibbs sampling randomly walks through the parameter space. Constructing a Markov chain using Gibbs sampling [60] can occur iteratively on each dimension of ***θ***, (or a partition of dimensions (see Section IV-A3)), conditional on the most recent values of the other dimensions. Denoted by the superscript, at the *m*th iteration of the MCMC simulation, random samples are proposed from all conditional distributions:

(8a) $$p\left(\theta_{1}^{\left(m\right)}|\theta_{2}^{\left(m-1\right)},\ldots ,\theta_{D}^{\left(m-1\right)},\text{y}\right),$$

(8b) $$p\left(\theta_{2}^{\left(m\right)}|\theta_{1}^{\left(m\right)},\ldots ,\theta_{D}^{\left(m-1\right)},\text{y}\right),$$

(8c) …,

(8d) $$p\left(\theta_{D}^{\left(m\right)}|\theta_{1}^{\left(m\right)},\theta_{2}^{\left(m\right)},\ldots ,\text{y}\right).$$

If the conditional distributions are known, a high-dimensional problem can be broken down into a sequence of smaller, low-dimensional conditional simulations. If, however, the conditional distributions are not known, other methods such as the MHRW will need to be used. The Gibbs sampler is a special case of MHRW where ***θ***′ is always accepted due to the acceptance probability ratio in (6) being one.

**Figure 2:**
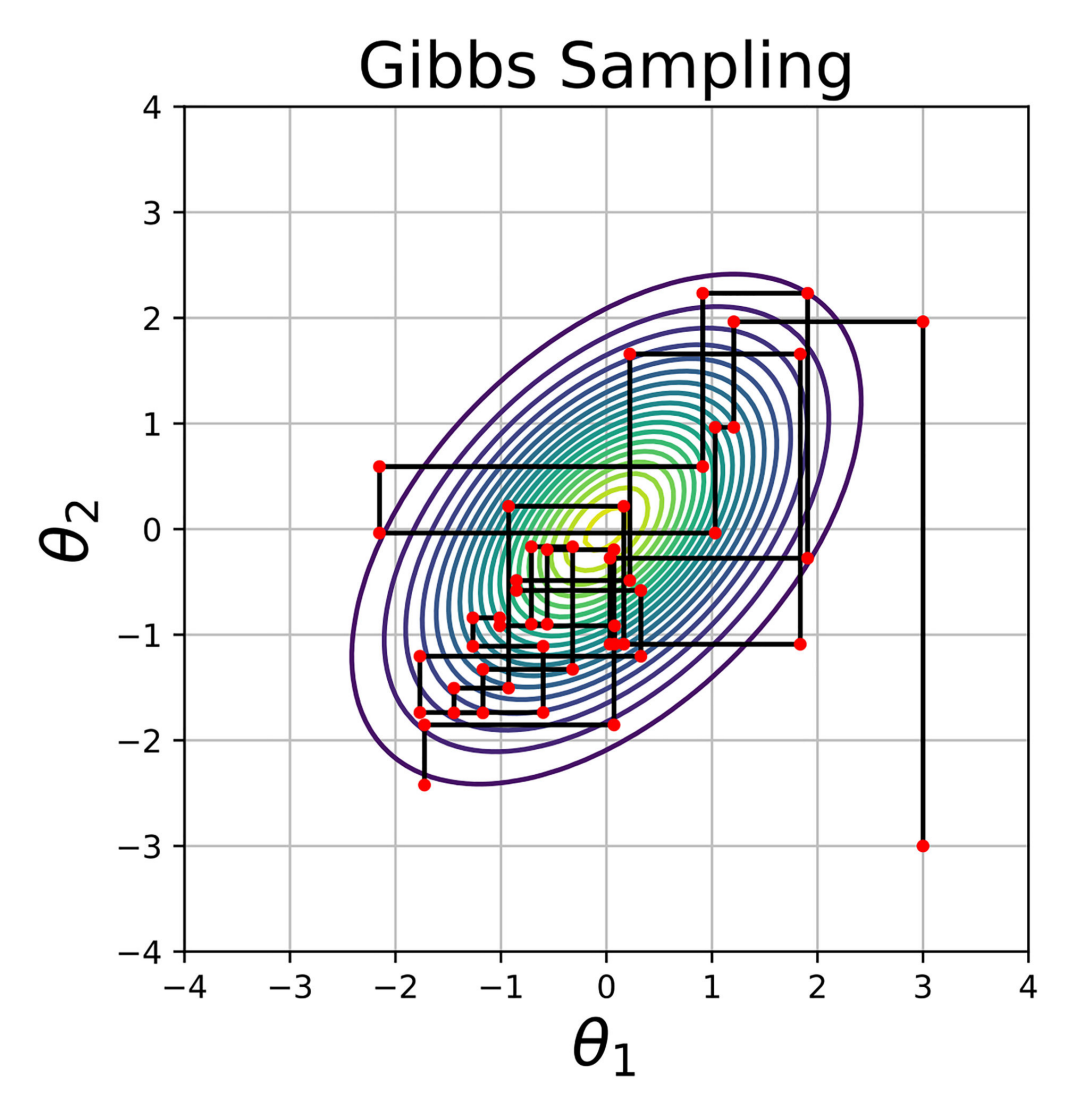
Sampling a 2-dimensional Gaussian distribution with Gibbs with 0 means for *θ*_1_ and *θ*_2_ for 50 MCMC iterations. The solid black lines show the trajectory between two successive accepted samples represented by the red dots.

Two advantages of Gibbs sampling over MHRW are not having to define a proposal distribution and proposals always being accepted. However, to employ Gibbs sampling, the conditional distributions need to be derived which can be problematic. The efficiency of Gibbs sampling can also be a concern if the dimensions of ***θ*** are highly correlated. Figure 2 shows an example where the Gibbs sampler struggles to sample efficiently from a 2-dimensional Gaussian distribution.

Gibbs sampling is particularly suitable for calibrating complex hierarchical Bayesian models, as discussed in Section VI-B. These models can be broken down into smaller sub-models, and Gibbs sampling facilitates the iterative sampling of these sub-models. If conjugacy exists for a subset of parameters within the model, Gibbs sampling can be employed for those conjugate parameters. An example illustrating this can be found in [24], where the transmission parameter for *Staphylococcus aureus* has a Gamma prior, which is conjugate to the Binomial likelihood.

### 3) Metropolis Within Gibbs

As explained in Section IV-A2, the Gibbs sampler is suitable when the conditional distributions of the posterior are known. However, when dealing with challenging conditional distributions, a practical approach is to employ a MH step to sample a subset of parameters for which the conditionals are not known, resulting in block updates. This method is known as the Metropolis Within Gibbs sampler [61] and proves to be an attractive option when modeling AMR [26], [28], [33], [38], [50], [53].

### 4) Delayed Rejection Adaptive Metropolis

The need to fine-tune the proposal e.g. : Σ in Equation (5) can be time-consuming, given its problem-specific nature. Adaptive MCMC [62] and the Delayed Rejection Adaptive Metropolis (DRAM) algorithm [63], [64] address this challenge by enabling automatic adjustments of exploration strategies. This adaptability, grounded in the characteristics of the data and model complexity, significantly improves the efficiency and effectiveness of parameter estimation. This is particularly valuable in scenarios like AMR, where disease dynamics are influenced by various factors and exhibit nonlinear behavior [18], [19], [57].

### 5) Hamiltonian Monte Carlo

As discussed in previous sections, MHRW, Gibbs and DRAM samplers explore *π*(***θ***) by randomly proposing a new state ***θ***′ from the current state ***θ*** using some proposal distribution. In high-dimensional problems, this random exploration can be inefficient. Hamiltonian Monte Carlo (HMC) addresses this issue by generating new proposal samples based on gradient information about *π*(***θ***), encouraging greater exploration of the parameter space. HMC was first developed in the late 1980s [65] and has gained popularity in the last decade as an effective approach for implementing MCMC [66], [67].

**Figure 3:**
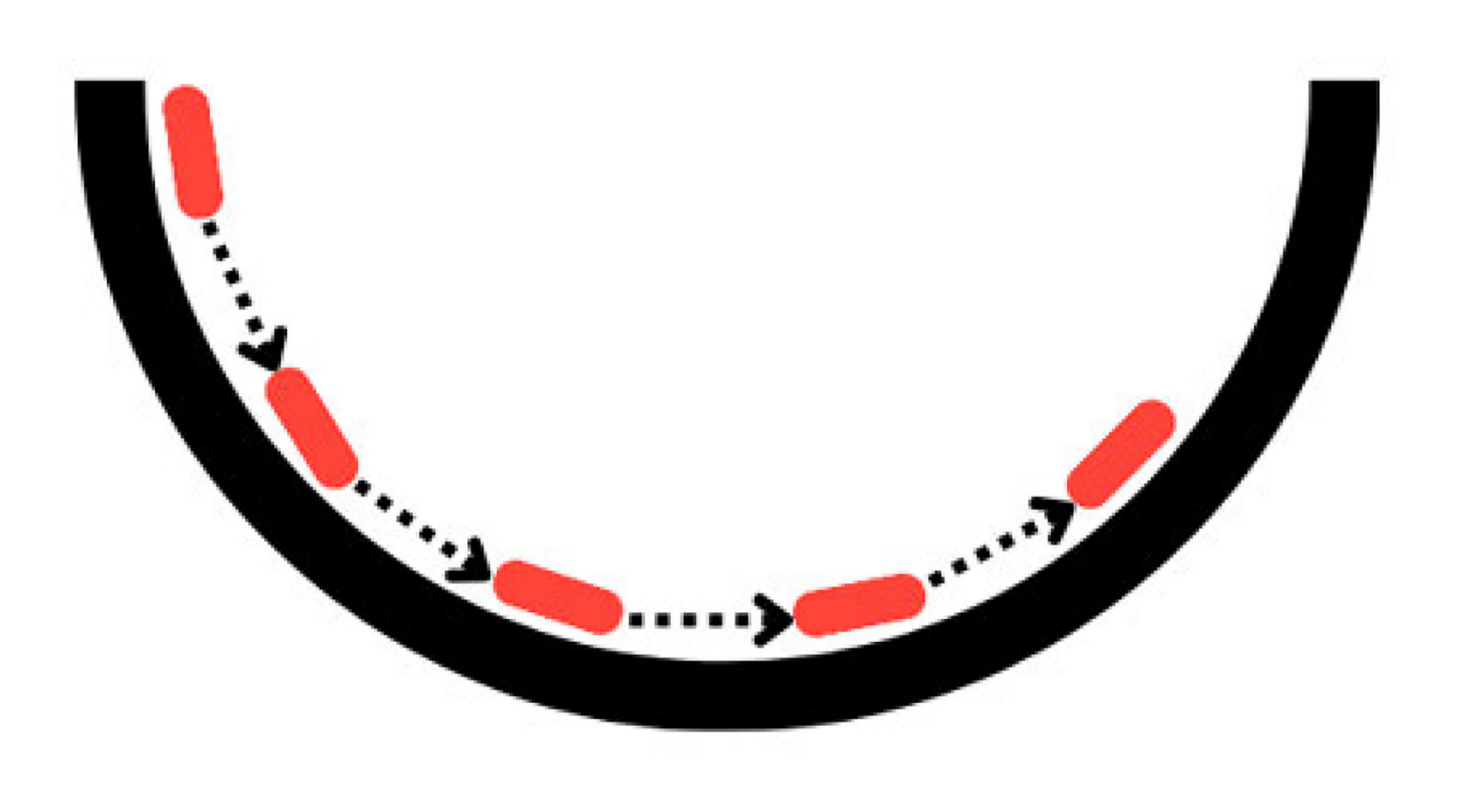
HMC analogy: The starting position of the left most puck (red) in a frictionless bowl (black solid line) will have potential energy but no kinetic energy. As the puck follows the trajectory of the arrows, potential energy is changed to kinetic energy.

HMC simulates a trajectory from ***θ*** to ***θ***′ by employing Hamilton’s equations. Conceptually, Hamilton’s equations represent a frictionless puck moving on a surface, such that the potential energy is defined as the negative logarithm of *π*(***θ***), *U*(***θ***) = − log(*π*(***θ***)). Figure 3 outlines this phenomenon. The surface is analogous to *π*(***θ***) which can have hills and valleys. The height of the surface corresponds to the potential energy function of the system. The higher the point on the surface, the higher the potential energy. The puck’s kinetic energy, *K*(**r**), corresponds to the energy associated with the momentum of the “puck” which is denoted **r**. The faster the puck is moving, the higher its kinetic energy. The total energy of the system is the sum of its potential and kinetic energies *H*(***θ***, **r**) = *K*(**r**) + *U*(***θ***). In Hamiltonian dynamics, the total energy remains constant over time, as long as there are no external forces acting on the system (i.e., in our frictionless scenario). Hamiltonian dynamics describe how the puck’s position and momentum change over time. These changes are governed by Hamilton’s equations, which depend on the gradient of the potential energy (surface) ∇*U*(***θ***) and the momentum of the system. Hamilton’s equations are defined as:

(9) $$\frac{d\theta }{dt}=\frac{\partial H(\theta ,\text{r})}{\partial \text{r}},\;\frac{d\text{r}}{dt}=-\frac{\partial H(\theta ,\text{r})}{\partial \theta }.$$

The joint density over ***θ*** and **r** is:

(10) p(θ,r)∝exp(−H(θ,r))

(11) =exp(−K(r))⋅exp(−U(θ))

(12) =p(θ)p(r).

As *θ* and **r** are independent in (12), **r** can be sampled from any distribution. For simplicity, this is often chosen to be Gaussian with zero mean.

A numerical integrator is necessary to discretize the differential equations in (9). The leapfrog numerical integrator is widely used as it is reversible and therefore maintains detailed balance. The leapfrog step alternates between updating the position of the puck based on its momentum and updating its momentum based on the gradient of the potential energy. Simulating a leapfrog trajectory with some momentum **r** causes ***θ*** to transition to ***θ***′. When starting at ***θ***′, if the same momentum is negated to −**r, *θ*** ′ follows the same trajectory back to ***θ***. A single iteration of HMC consists of *L* leapfrog steps:

(13) $${\text{r}}^{\prime }=\text{r}+0.5\cdot \epsilon \cdot \nabla U(\theta ),$$

(14) $$\theta^{\prime }=\theta +\epsilon \cdot {\text{r}}^{\prime },\;$$

(15) $${\text{r}}^{\prime }={\text{r}}^{\prime }+0.5\cdot \epsilon \cdot \nabla U\left(\theta^{\prime }\right),$$

where *ϵ* is the step size. The acceptance criterion for HMC is defined as the following special case of (6):

(16) $$\alpha \left(\theta ,\theta^{\prime }\right)=\text{min}\left\{1,\frac{\text{exp}\left\{U\left(\theta^{\prime }\right)-\frac{1}{2}{\text{r}}^{\prime }\cdot {\text{r}}^{\prime }\right\}}{\text{exp}\left\{U(\theta )-\frac{1}{2}\text{r}\cdot \text{r}\right\}}\right\}.$$

**Figure 4:**
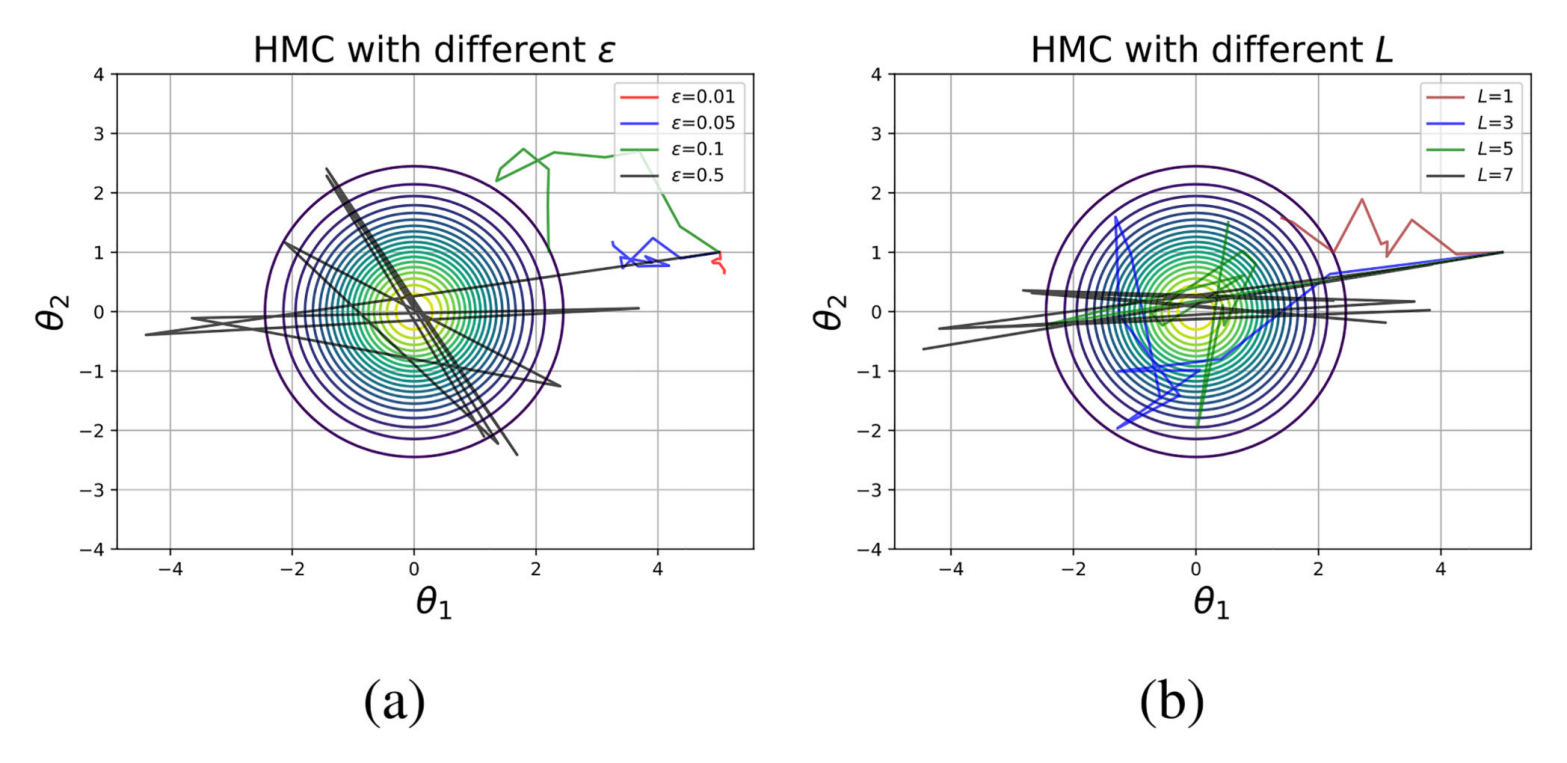
Sampling a 2-dimensional Gaussian distribution with HMC when (a) changing *ϵ* and *L* = 5 and (b) changing parameter *L* with *ϵ* = 0.1 for 10 MCMC iterations.

Simulating the dynamics of the system using Hamiltonian dynamics and leapfrog integration and employing the acceptance step in (16), we can sample from *π*(***θ***). The sampled positions of the puck correspond to samples from *π*(***θ***). The samples generated are governed by a predefined number of steps *L* of size *ϵ*, determined by the user. The choice of *L* and *ϵ* is problem specific as HMC is sensitive to the choice of these parameters. If *L* is too large, computation time can be wasted, as the trajectory might end up close to where it started. On the other hand, if *L* is too small, the proposal may exhibit random-walk behavior. Figure 4 illustrates an example when sampling from a 2-dimensional Gaussian distribution. Figure 4(a) and (b) depict how the choice of step-size and parameter *L*, respectively, can impact the efficiency of the sampler.

### 6) Metropolis Hastings Langevin Algorithm

The Metropolis Adjusted Langevin Algorithm (MALA) [68] can be regarded as a specific instance of HMC when *L* = 1. In MALA, the gradient of the posterior ∇*π*(***θ***) is integrated into the Metropolis proposal, serving as a drift term that proposes ***θ***′ in regions of higher probability within *π*(***θ***). An illustration of this approach can be found in [38], where parameters are estimated using both a Metropolis Within Gibbs step and a standalone Metropolis step through the modified Langevin-Hastings algorithm when modeling MIC distributions of *E. coli* tested against ampicillin. The proposal is defined to be

(17) $$\theta^{\prime }=N\left(\theta^{\prime };\theta +\frac{1}{2}\text{Γ}\nabla \pi (\theta ),\text{Γ}\right),$$

where **Γ** = *γ* ^2^***I***_*d*_, for step size *γ*. Selecting an appropriate step size for MALA involves a trade-off between exploration and exploitation, and it often requires experimentation and fine-tuning based on the characteristics of *π*(***θ***) and the problem at hand.

### 7) No-U-Turn Sampler

The No-U-Turn Sampler (NUTS) [69] is an extension of HMC which adaptively estimates *L*. NUTS underpins the probabilistic programming languages (ppls) Stan [70], PyMC3 [71] and Pyro [72] and has been used in a variety of AMR studies [37], [41], [44], [45], [51].

### 8) Reversible Jump - Markov Chain Monte Carlo

Sampling with MHRW, Gibbs, HMC and NUTS assumes that the probability distribution *π*(***θ***) has a fixed number of dimensions. In some scenarios, there might be multiple competing models that could be applicable within the defined framework. These models could differ in aspects such as dimensions or interpretations of the likelihood function. For instance, consider count data like the number of vancomycinresistant *enterococci* in a hospital ward [73]; such data could be modeled using a Negative Binomial or Poisson likelihood, depending on the over-dispersion of the count data. Employing MHRW would require conducting independent MCMC runs for each candidate model. Reversible Jump MCMC (RJ-MCMC) [74] is a MHRW-based algorithm designed to sample from *π*(*D*, ***θ***_*D*_|**y**) by jumping between competing models as part of the sampling process. The jump is crafted to maintain detailed balance, ensuring the algorithm’s validity. An application of RJ-MCMC can be found in the analysis of nosocomial infection data [53]. In this model, the unobservable times at which patients acquire a drug-resistant organism and the total number of acquisitions are both unknown. Since the dimensionality of the model can change, RJ-MCMC proves to be a suitable method for sampling from the joint distribution of all the model unknowns.

### B Rejection and Importance Sampling

The MCMC methods discussed in Section IV-A are Monte Carlo techniques that build a Markov chain by drawing samples from *π*(***θ***). Characteristics of *π*(***θ***), such as its mean and variance, can be approximated using the Markov chain. However, when sampling directly from *π*(***θ***) is challenging, other Monte Carlo methods like importance sampling and rejection sampling can be employed. Both importance and rejection sampling involve transforming samples drawn from a proposal distribution *q*(***θ***) into samples from *π*(***θ***). The choice of *q*(***θ***) can be a practical challenge, especially in high-dimensional spaces, but it can be any distribution that is evaluable at different points.

#### 1) Rejection Sampling

In rejection sampling, a constant *c* and proposal distribution *q*(***θ***) needs to be chosen such that:

(18) cq(θ)>π(θ),∀θ.

The idea is that a sample from *q*(***θ***) can be used when accepting a sample from *π*(***θ***) with probability $\frac{\pi (\theta )}{cq(\theta )}$. Samples are generated from *q*(***θ***) until ***θ*** is accepted. This should lead to more samples around ***θ*** being accepted when *π*(***θ***) is relatively large and *q*(***θ***) is relatively small. In contrast, a relatively small *π*(***θ***) and large *q*(***θ***) will result in more samples of ***θ*** being rejected. The accept/reject step is similar to that as described in the MHRW algorithm (see Section IV-A1). A random variable *u* is drawn from a Uniform distribution on [0, 1] such that if *u* is smaller than the acceptance probability, $u<\frac{\pi (\theta )}{cq(\theta )}$, ***θ*** will be rejected.

It can, however, be problematic finding a suitable *c* and *q*(***θ***) such that a high percentage of samples are accepted and *cq*(***θ***) *> π* (***θ***), ∀***θ***.

#### 2) Importance Sampling

Importance sampling overcomes this by weighting samples and providing an estimator for the expectation of *π*(***θ***) by

(19) $$E[\pi (\theta )]\approx \frac{1}{N}\sum_{i=1}^{N}\frac{\pi \left(\theta_{i}\right)}{q\left(\theta_{i}\right)}.$$

The weights in IS represent a scaled version of the rejection probabilities and serve to quantify the likelihood that sampled values from *q*(***θ***) originate from *π*(***θ***).

Although these two methods are not used to calibrate models to data in the studies identified in this review, they form the basis of the calibration methods described in the subsequent sections.

### C Approximate Bayesian Calibration

The definition of the likelihood in Bayes’ theorem (2) is crucial as it encapsulates the probability of the data originating from the model. In certain scenarios, defining the likelihood may be challenging or computationally intensive, especially when calibrating stochastic compartmental models (CMs) or ABMs [13], [20], [21], [30], [48]. Approximate Bayesian Computation (ABC) involves simulating data from the model with a set of parameters ***θ***. A summary statistic, such as the root mean square error (RMSE) or Kullback-Leibler (KL) divergence, quantifies the closeness between the simulated and observed data. Numerous sets of parameters ***θ*** are simulated from the prior distribution and assessed against the true observations. The summary statistic acts as a pseudo-likelihood, helping distinguish between different parameter sets. ABC-rejection sampling [13], [20], [30], [48] follows a similar procedure as rejection sampling in Section IV-B. A user-defined tolerance or threshold determines whether a particular ***θ*** is accepted or rejected.

**Figure 5:**
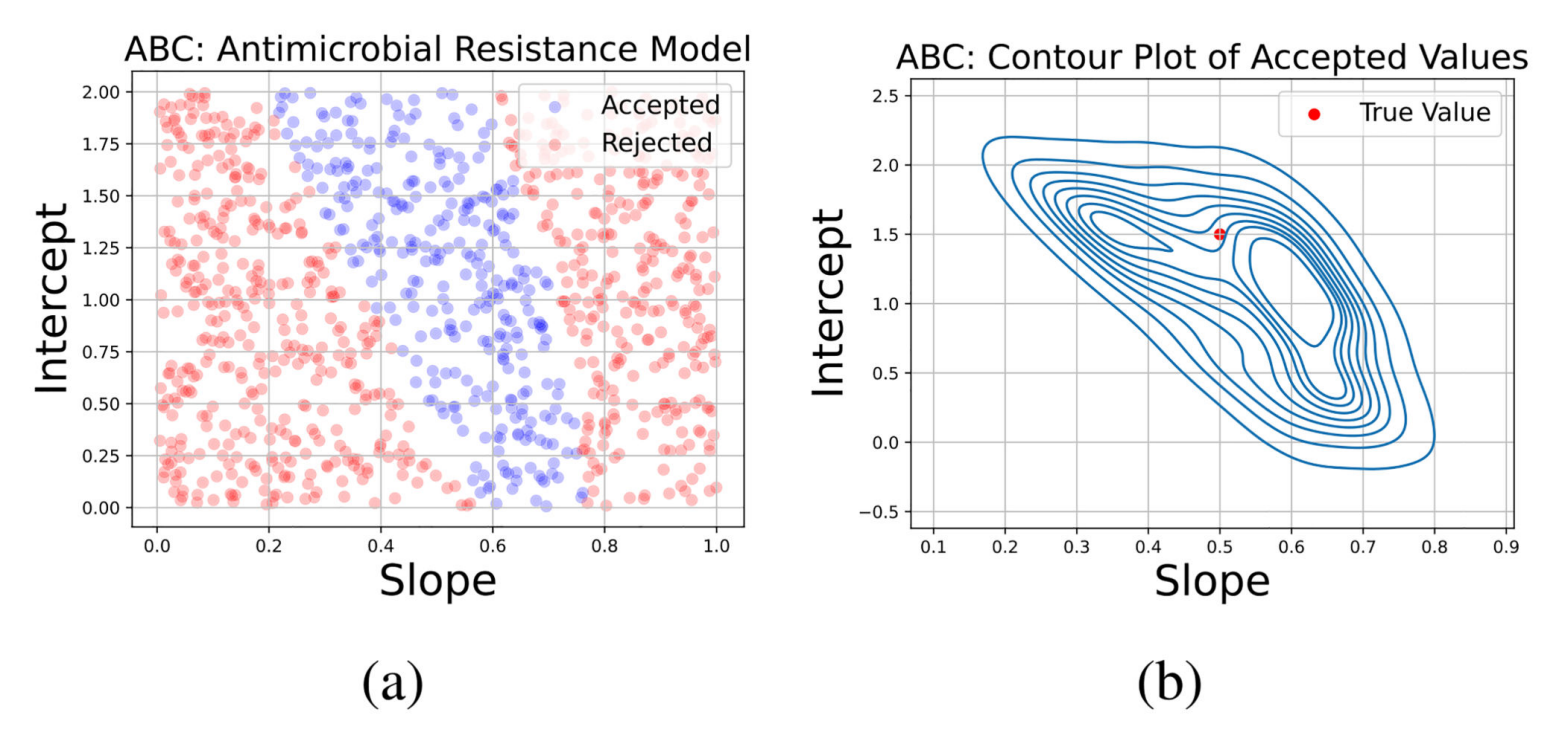
Toy example of using approximate Bayesian computation to estimate the parameters governing the linear relationship between antibiotic concentration and bacterial growth inhibition. (a) The blue and red points are the accepted and rejected values, respectively. (b) Is the contour plot of the accepted values with the true set of values indicated by the red dot.

An example of ABC applied to a simple AMR related problem can be seen in Figure 5. The example considers estimating the relationship between antibiotic concentration and bacterial growth inhibition, which is a linear relationship. Firstly, data is generated from the simple linear model with slope and intercept parameters fixed at 0.5 and 1.5, with Uniform [0, 1] and [0, 2] priors, respectively. The ABC algorithm samples from the parameter space by simulating data using sampled parameters form the prior and calculates the summary statistic, which is the Mean Absolute Error (MAE), between the distance between simulated and observed data. Parameters are then accepted if the distance is below a certain tolerance level. Figure 5 (a) depicts the parameter sets that are within the tolerance threshold (blue) and the rejected parameter sets (red). A contour plot of the accepted values can then be seen in Figure 5 (b).

Examples of threshold determination in the context of AMR include simulated *M. tuberculosis* incidence falling within the 95% confidence interval of the true observations [21] or a value resulting in the closest 2.5 or 5% of simulations to the targets not being rejected [48]. Parameters leading to pseudo-likelihoods smaller than a given tolerance constitute the posterior distribution, while those exceeding it are rejected. ABC-rejection sampling may be inefficient if the prior does not closely resemble the posterior (e.g., due to a very uninformative prior) or if the model is high-dimensional, requiring numerous simulations for a high acceptance rate. Intelligent methods for selecting ***θ***, such as Latin Hypercube Sampling [21], exist to address this issue.

### D State-Space Models

State-space models (SSMs) or Hidden Markov Models (HMMs) (often used interchangeably) are commonly employed in time-series problems to represent the probabilistic dependence at time-step *t* between the hidden (or unobservable) states of a model, denoted as **X**_*t*_, and observed variables, denoted as **Y**_*t*_. SSMs model how the state **X**_*t*−1_ is transitioned to **X**_*t*_ through the linear or nonlinear function *f*_*t*_(). An observation function, *g*_*t*_(), illustrates the relationship between the dynamical model and the observations. A stochastic formulation is presented as follows:

(20) $${\text{X}}_{t}\sim f_{t}\left({\text{X}}_{t-1},V_{t}\right),$$

(21) $${\text{Y}}_{t}\sim g_{t}\left({\text{X}}_{t},W_{t}\right),$$

where the initial state at time 0 is defined to be *x*_0_ and is drawn from the distribution *µ*(), and *V*_*t*_ and *W*_*t*_ are independent and identically distributed process noise. A deterministic representation of an SSM is equivalent to (20) and (21) without the process noise.

#### 1) Example of a Deterministic SSM

**Figure 6:**
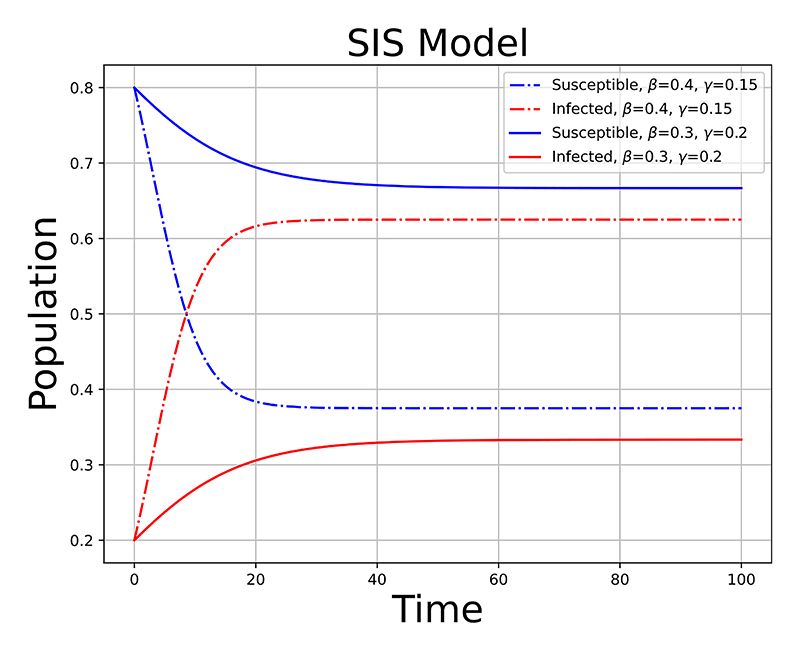
Two simulations of the discrete time approximation of the ODEs in (22) and (23) when *β* = 0.4, *γ* = 0.15 (dashed/dot lines) and *β* = 0.3, *γ* = 0.2 (solid lines).

An example of an SSM, which is related to AMR epidemiology, is the Susceptible, Infected, Susceptible (SIS) disease model, a variant of the more widely known Susceptible, Infected, Recovered (SIR) model [75]. The key distinction lies in the assumption that individuals do not gain immunity after recovering from infection in the SIS model. This makes the SIS model particularly relevant when modeling scenarios related to AMR, where individuals can become repeatedly infected with the same pathogen or variant. Some examples include: *P. aeruginosa* [50]; beta-lactamase producing *E. coli* and *K. pneumoniae* [36]; *A. baumannii* [46] and *N. gonorrhoea* [41]. A common approach to representing SSMs are Ordinary differential equation (ODE) [16], [18], [35]. A discrete time approximation of ODEs for the SIS model are defined as

(22) $$S_{t+1}=S_{t}-\frac{\beta I_{t}S_{t}}{P_{op}}-\gamma I_{t}\text{Δ}t,$$

(23) $$I_{t+1}=I_{t}+\frac{\beta I_{t}S_{t}}{P_{op}}+\gamma I_{t}\text{Δ}t,$$

where *β* and *γ* are the effective transmission rate of the disease and the mean recovery rate, respectively, Δ*t* is the interval between timesteps and *P*_*op*_ is the total population. In the context of AMR, a population could signify individuals in a homogeneous population that originate in a susceptible compartment and become colonised then infected by a drug resistant pathogen [16], or genetic plasmids encoding enzymes that confer resistance to antibiotics [35]. The parameters *β* and *γ* govern how much of the population get infected at each increment of time. This is summarised by the reproductive number, *R*, which is calculated by *R* = *β/γ*. Figure 6 exemplifies how the *R* number can effect the total number of individuals in the susceptible and infected compartments when *R* = 2.66 (dashed/dot lines) and *R* = 1.5 (solid lines). Equations (22) and (23) are analogous to (20) such that **X**_*t*_ = {*S*_*t*_, *I*_*t*_}. In the context of AMR modelling, examples of the observation equation in (21), often referred to as the likelihood, are the Binomial distribution [46], Bernoulli distribution [36] or beta-binomial and dirichlet-multinomial distributions [41].

### E Sequential Monte Carlo Methods

#### 1) Particle Filter

To formulate the problem, consider an SSM that is simulated for *t* timesteps, where data is acquired at each increment of time. For brevity, the sequence of states and observations up to time *t* can be defined as **x**_0:*t*_ = {*x*_0_, *x*_1_, …, *x*_*t*_} and **y**_0:*t*_ ={*y*_0_, *y*_1_, …, *y*_*t*_}, respectively. In filtering problems, the posterior distribution *p*(**x**_0_:_*t*_| **y**_1_:_*t*_, ***θ***) can be estimated recursively by prediction-update steps. Assuming Markovianity, the posterior can be defined as *p*(**x**_*t*_ |**y**_1:*t*_, ***θ***). The posterior from the previous timestep, *p*(**x**_*t*−1_ |**y**_1:*t*−1_, ***θ***), and the state equation in (20) can be used to predict **x**_*t*_. This is done by evaluating *p*(**x**_*t*_ |**y**_1_:_*t* −1_, ***θ***) by using the Chapman-Kolmogorov equation such that

(24) $$\begin{array}{l} p\left(x_{t}|y_{1:t-1},\theta \right)\\ \;\;=\int p(x_{t}|x_{t-1},\theta )p(x_{t-1}|y_{1:t-1},\theta )dx_{t-1}, \end{array}$$

(25) $$=\int p\left({\text{x}}_{t}|{\text{x}}_{t-1},\theta \right)p\left({\text{x}}_{t-1}|{\text{y}}_{1:t-1},\theta \right)d{\text{x}}_{t-1},$$

where *p*(**x**_*t*_|**x**_*t*−1_, ***θ***) is equal to *p*(**x**_*t*_|**x**_*t*−1_, **y**_1_:_*t* −1_, ***θ***) because (20) is a Markov process of order one. As data at timestep *t* becomes available, the prediction step can be updated using Bayes’ theorem in (1) such that the posterior is defined to be

(26) $$p\left({\text{x}}_{t}|{\text{y}}_{1:t},\theta \right)=\frac{p\left({\text{y}}_{t}|{\text{x}}_{t},\theta \right)p\left({\text{x}}_{t}|{\text{y}}_{1:t-1},\theta \right)}{p\left({\text{y}}_{t}|{\text{y}}_{1:t-1},\theta \right)}$$

(27) $$=\frac{p\left({\text{y}}_{t}|{\text{x}}_{t},\theta \right)p\left({\text{x}}_{t}|{\text{y}}_{1:t-1},\theta \right)}{\int p\left({\text{y}}_{t}|{\text{x}}_{t},\theta \right)p\left({\text{x}}_{t}|{\text{y}}_{1:t-1},\theta \right)d{\text{x}}_{t}}.$$

If the integrals in (25) and (26) are high dimensional, they can be hard to solve analytically. Section IV-A outlines how MCMC can sample from complex distributions that are static. However, as the posterior distribution is dynamic and needs to be estimated recursively, MCMC methods are unsuitable. One method to overcome this issue is to approximate (25) and (26) recursively using a particle filter.

Particle filters are an SMC method which uses the IS principles outlined in Section IV-B in a recursive process to infer the time-dependent hidden states in SSMs that are nonlinear and non-Gaussian [76]. At every timestep *t*, the particle filter draws *N* samples (particles) from a proposal distribution, *q* (**x**_1_:_*t*_|_*t*_ **y**_1_:_*t*_), which is parameterised by the sequence of states and observations. The samples are statistically independent and each represents a different hypothesis of the sequence of states of the system. The *i*th sample has an associated weight, ${\text{w}}_{t}^{i}$, which indicates the relative importance of each of the corresponding samples. A set of *N* particles can then be represented as ${\left\{{\text{x}}_{1:t}^{i},{\text{w}}_{t}^{i}\right\}}_{i=1}^{N}$.

By following (19), the weights of the particles can be calculated by

(28) $${\text{w}}_{1:t}^{i}=\frac{p\left({\text{x}}_{0:t}^{i},{\text{y}}_{1:t}\right)}{q\left({\text{x}}_{0:t}^{i}|{\text{y}}_{1:t}\right)}.$$

As is with the case for (26), the state at timestep *t* is required (not the full state sequence), such that (28) can be rewritten as

(29) $${\text{w}}_{1:t}^{i}={\text{w}}_{1:t-1}^{i}\frac{p\left({\text{y}}_{t}|{\text{x}}_{t}^{i},\theta \right)p\left({\text{x}}_{t}^{i}|{\text{x}}_{t-1}^{i},\theta \right)}{q\left({\text{x}}_{t}^{i}|{\text{x}}_{t-1}^{i},{\text{y}}_{t},\right)},$$

where ${\text{w}}_{1:t-1}^{i}$ is the weight from the previous timestep. For *t* = 1,

(30) $${\text{w}}_{1}^{i}=\frac{p\left({\text{y}}_{1}|{\text{x}}_{1}^{i},\theta \right)p\left({\text{x}}_{1}^{i}|\theta \right)}{q\left({\text{x}}_{1}^{i}|{\text{y}}_{1}\right)}.$$

The normalised weights can be computed by

(31) $${\hat{w}}_{1:t}^{i}=\frac{{\text{w}}_{1:t}^{i}}{\sum_{i=1}^{N}{\text{w}}_{1:t}^{i}},$$

which can be used to calculate an estimate of the true state **x**_*t*_ via a weighted sum:

(32) $$E\left({\text{x}}_{t}\right)=\sum_{i=1}^{N_{x}}{\text{x}}_{t}^{i}{\hat{\text{w}}}_{1:t}^{i}$$

The algorithm described up to now is termed Sequential Importance Sampling (SIS). As time evolves, the normalised weights in (31) can suffer from the phenomenon particle degeneracy. This occurs when one weight becomes close to one while the remainder tend to 0. To overcome this, a process reminiscent to the survival of the fittest can be employed. Calculating the number of effective samples:

(33) $$N_{eff\;}=\frac{1}{\sum_{i=1}^{N}{\left({\tilde{\text{w}}}_{1:t}^{i}\right)}^{2}},$$

at each iteration can help diagnose when particle degeneracy is occuring. Once *N*_*eff*_ drops below a certain threshold, resampling can be used to replicate particles with higher weights and kill off particles with lower weights. Multinomial resampling is commonly used which draws a new set of *N* particles based on the current set and proportionally to their normalised weights

(34) $${\tilde{\text{w}}}_{1:t}^{i}\;\text{for}\;i=1,\ldots ,N.\;$$

After resampling, the normalised weights and unnormalized weights are set to $\frac{1}{N}$ and $\frac{1}{N}\sum_{i=1}^{N}{\text{w}}_{1:t}^{i}$, respectively.

Stochastic elements within a model can result in particles that have slightly different representations of the model. This makes the PF a suitable candidate when modelling stochastic SSMs. Table 2 outlines the limited number of published studies that use a PF to model stochastic SSMs relating to AMR [36], [39], [59]. A potential reason for this low number is that the PF can be computationally expensive to run if there are lots of particles. An estimate of the log-likelihood from the particle filter can be used within the inference algorithms p-MCMC [39], [59] and SMC^2^ [36]. The log-likelihood calculation is outlined in Section IV-E2.

#### 2) Particle - Markov-Chain Monte Carlo

P-MCMC obtains numerical estimates related to *π*(***θ***) by combining two Monte Carlo methods. In the original contribution of [77], an outer MCMC layer is used to estimate the parameters of an SSM while an inner PF layer estimates the states of the dynamical system. The outer MCMC layer runs in a similar fashion to the MHRW algorithm in Section IV-A1, but the log-likelihood is estimated by the PF in Section IV-E1. For *t* = 1, .., *T* an unbiased estimate of the log-likelihood can be estimated recursively by summing the log-weights:

(35) $$p\left({\text{y}}_{1:T}|\theta \right)\approx \frac{1}{N}\sum_{i=1}^{N}{\text{w}}_{1:t}^{i}.$$

This is a byproduct of running the PF and so no additional calculations are required. The resulting log-likelihood estimate can be used within the acceptance probability in (6) such that

(36) $$\alpha \left(\theta^{\prime },\theta \right)=\text{min}\left\{1,\frac{\pi \left(\theta^{\prime }\right)q\left(\theta |\theta^{\prime }\right)}{\pi (\theta )q\left(\theta^{\prime }|\theta \right)}\right\},$$

where *π*(***θ***) is the product of the prior density *p*(***θ***) and the approximate likelihood *p*(**y**_1:*T*_ |***θ***). As with the MHRW algorithm, when a symmetric proposal for ***θ*** is used, (36) cancels to

(37) $$\alpha \left(\theta^{\prime },\theta \right)=\text{min}\left\{1,\frac{\pi \left(\theta^{\prime }\right)}{\pi (\theta )}\right\}.$$

Reference [77] proves that the Markov chain converges to *π*(***θ***) when using a fixed number of particles within the PF.

Two examples of utilizing P-MCMC to infer the parameters of models related to antibiotic resistance in *N. gonorrhoea* can be found in [39] and [59]. A disadvantage of P-MCMC is that it can be impractical to run if one iteration of the Particle Filter (PF) is computationally expensive. Due to the sequential nature of P-MCMC and MCMC algorithms, parallelization over the *M* MCMC iterations is not readily available. SMC samplers and SMC^2^, described in Sections IV-E3 and IV-E4, respectively, are two alternatives to MCMC and P-MCMC, which can easily exploit high-performance computing architectures. Although IS sampling in the PF can be readily parallelised, we note that the computational bottleneck occurs when estimating the log-likelihood from a PF. Therefore, parallelising the instances of the PFs is more desirable.

#### 3) Sequential Monte Carlo Samplers

Sequential Monte Carlo (SMC) samplers [78], [79] leverage the principles of both MCMC and IS to sequentially sample from *π*(***θ***). At each iteration *k*, SMC samplers provide approximations for intermediate target distributions of interest, *π*_1_(***θ***_1_), …, *π*_*K*_ (***θ***_*K*_), ultimately converging to the terminal distribution *π*_*K*_ (***θ***_*K*_), which accurately represents *π*(***θ***). The joint distribution encompassing all intermediate target distributions is formulated as follows:

(38) $$\pi_{K}\left(\theta_{1:K}\right)=\pi_{K}\left(\theta_{K}\right)\prod_{k=2}^{K}L\left(\theta_{k-1}|\theta_{k}\right).$$

where *L*(***θ***_*k*−1_ |***θ***_*k*_) denotes the L-kernel. The L-kernel is a probability distribution defined by the user and plays a crucial role in determining the efficiency of the sampler [80].

The initialization step of the Sequential Monte Carlo (SMC) sampler involves using importance sampling. At *k* = 1, *N* statistically independent samples are drawn from a prior distribution *q*_1_(·) according to:

(39) $$\theta_{1}^{i}\sim q_{1}(\cdot ),\;\forall i.$$

Following (19), each sample is assigned an importance weight using the following formula:

(40) $${\text{w}}_{1}^{i}=\frac{\pi \left(\theta_{1}^{i}\right)}{q_{1}\left(\theta_{1}^{i}\right)},\;\forall i.$$

where $\pi_{1}\left(\theta_{1}^{i}\right)$ is the prior distribution, and $q_{1}\left(\theta_{1}^{i}\right)$ is the proposal distribution at time step *k* = 1.

As time evolves, samples are proposed based on the previous iteration. Equation (5) represents a commonly chosen proposal distribution for SMC samplers:

(41) $$\theta_{k}^{i}\sim q\left(\theta_{k}^{i}|\theta_{k-1}^{i}\right).$$

After new samples are proposed, they are assigned weights according to:

(42) $${\text{w}}_{k}^{i}={\text{w}}_{k-1}^{i}\frac{\pi \left(\theta_{k}^{i}\right)}{\pi \left(\theta_{k-1}^{i}\right)}\frac{L\left(\theta_{k-1}^{i}|\theta_{k}^{i}\right)}{q\left(\theta_{k}^{i}|\theta_{k-1}^{i}\right)},\;\forall i.$$

Similar to the PF, following the importance sampling step, the weights are normalized according to (31), and resampling is performed if the number of effective samples *N*_*eff*_ in (33) falls below a certain threshold. This process is repeated for *K* iterations, and in contrast to PFs, it is assumed with SMC samplers that all data is known before each run. In a similar fashion to (32), estimates of the expectations of functions are obtained by

(43) $$E\left(f\left(\theta_{k}^{i}\right)\right)=\sum_{i=1}^{N}{\tilde{\text{w}}}_{k}^{i}\theta_{1:k}^{i}.$$

In contrast to using samples from the previous iteration only, as is with (43), a process termed recycling [80], [81] can be employed which provides estimates using all samples from all iterations. Let

(44) $${\text{c}}_{k}=\frac{{\text{l}}_{k}}{\sum_{k=1}^{K}{\text{l}}_{k}},\;\forall k,$$

where

(45) $${\text{l}}_{k}=\frac{{\left(\sum_{i=1}^{N}{\text{w}}_{k}^{i}\right)}^{2}}{\sum_{i=1}^{N}{\left({\text{w}}_{k}^{i}\right)}^{2}}.\forall k.$$

The expectation of functions can then be realised by

(46) $$E\left(f\left(\theta_{k}^{i}\right)\right)=\sum_{k=1}^{K}c_{k}E\left(f\left(\theta_{k}^{i}\right)\right),$$

where $\sum_{k=1}^{K}c_{k}=1$.

In situations where evaluating the likelihood in a SMC sampler is challenging or computationally expensive, it may be appropriate to utilize methods outlined in Section IV-C for simulating a pseudo-likelihood. This pseudo-likelihood can be employed in place of the actual likelihood. An illustration of this approach can be found in [43] when simulating the emergence of drug-resistant *N. gonorrhoea*.

#### 4) Sequential Monte Carlo^2^

Sequential Monte Carlo^2^ (SMC^2^) integrates the Particle Filter (PF) and Sequential Monte Carlo (SMC) samplers, as outlined in Sections IV-E1 and IV-E3, respectively, to estimate *π*(*θ*). Similar to p-MCMC, the PF yields an unbiased estimate of the likelihood (35), which can be incorporated into the target calculation of the SMC sampler. In (40), the target $\pi \left(\theta_{k}^{i}\right)$ is the product of the prior $p\left(\theta_{k}^{i}\right)$ and the likelihood $p\left({\text{y}}_{1:T}|\theta_{k}^{i}\right)$ (provided by the PF).

An example of SMC^2^ is presented in [36]. In this example, the likelihood and states of the model, which outline individuals colonized by drug-resistant bacteria, are estimated using the PF, while the SMC sampler estimates the model parameters.

## V Evaluating Bayesian Algorithms

When performing Bayesian analysis, it is essential to understand how well the proposed sampler is performing in terms of correctness, accuracy and efficiency. Critically evaluating outputs from different sampling techniques can help determine which sampler to use. This section outlines some of the methods provided in [82] on how to do this.

### A Recovering The Correct Distributions From The Model

Simulating data from a model with predefined parameters and subsequently testing the ability of the sampler to recover the correct parameters (posterior distribution) used in the data-generating process is a validated method to assess the accuracy of the sampling process [26], [28], [36], [50], [53].

Simulating data from the model and assessing their consistency with true observations through posterior predictive checks is a valuable method to determine if the model accurately represents reality [16], [19], [25], [27], [28], [30], [39], [46], [47], [49], [53], [59]. With MCMC methods, the posterior predictive distributions (PPD) manifest as a set of samples. To validate the credibility of the PPD, the true observations are expected to fall within the 95% confidence intervals of the samples, and the mean sample should follow the trend of the true observations. A goodness-of-fit test, such as the chi-squared test, can be employed for a visual inspection of the accuracy between the mean sample and true observations. An illustrative example is provided in [53], where the chi-squared test is used to compare the mean predicted numbers of positive swabs of vancomycin-resistant *enterococci* with the true observed swabs.

The practice of utilizing data to estimate the model and then comparing the results with the same data, often referred to as *using the data twice*, should be approached with caution to avoid potential biases. To address this concern, generating forecasts of true observations into the future, using data the model has not encountered, can be employed. An example of this approach is illustrated in Figure 2 of [39], where the model is fitted to annual *N. gonorrhoea* cases from 2008 to 2017, and forecasts are made for the period between 2018 and 2030. Simple scoring rules, such as those proposed by [83], can effectively summarize the statistical consistency between the PPD and the true observations. Notably, refinement and improvement of predictive performance for MCMC algorithms in forecasting COVID-19-related deaths are explored in [84] and [85].

Scoring rules are applicable when evaluating observable variables, such as positive swabs or colonized patients. However, for assessing latent quantities like the growth rate and reproductive number *R*_*t*_, simulation-based calibration (SBC) provides a valuable alternative [86]. In SBC, data is simulated from a model using parameters drawn from the prior distribution, and the posterior calibration over independent simulated datasets is then tested against the inference algorithm. An example of this approach is demonstrated in [59], where the total number of *N. gonorrhoea* cases and the number of cefixime-resistant infections are compared with simulated datasets generated using parameters sampled from their posterior distributions. This enables the assessment of the model’s goodness of fit to the data, with one simulation performed for each of the one thousand sampled parameter sets.

### B Evaluating Markov Chains

The MCMC sampling methods outlined in Section IV-A generate a Markov chain. It is essential to assess how effectively and efficiently a sampler is performing to ensure that the samples are correctly drawn from the posterior distribution. Evaluating Markov chains facilitates comparisons between different samplers and aids in deciding when to terminate the sampling process.

Using PPLs like Stan offers the advantage of automatic provision of summary statistics once the sampling process concludes. However, there are also standalone software packages that can be employed post-sampling. Two examples in the R programming language are CODA [87] and Bayesian Output Analysis (BOA) [88]. CODA has been utilized to evaluate chains sampled using MCMC for multidrug-resistant *A. baumannii* [47] and *N. gonorrhoea* [40], as well as p-MCMC for *N. gonorrhoea* [59]. Similarly, BOA has been applied in the analysis of *enterococci* [53]. These tools provide summary statistics such as effective sample size (ESS), autocorrelations, Gelman-Rubin statistics, among others. A number of commonly used statistics are described in the subsequent sections.

#### 1) Effective Sample Size

The effective sample size (ESS) serves as an explicit approximation of the number of independent samples necessary for the Markov chain to possess the same estimation power as the set of auto-correlated samples. Higher ESS values are desirable, indicating a more efficient sampler. Comparing ESS values per second of computation time can provide insights into the efficiency of different samplers. For example, MHRW runs may take less computation time than HMC and NUTS because MH does not involve gradient evaluations. The efficiency of a sampler can be assessed by calculating the ESS per second based on the time taken for the sampler to complete its run.

#### 2) Integrated Auto-Correlated Time

The Integrated Auto-Correlated Time (IACT) provides an estimate of the number of samples, on average, required to draw an independent sample from a continuous Markov chain. This measure is associated with the concept of “mixing” and lower IACT values are more desirable as they indicate faster mixing and better exploration of the parameter space.

#### 3) Gelman-Rubin Diagnostic

The Gelman-Rubin diagnostic [89] is a numerical method that determines if multiple chains have converged by comparing the variances between chains. This diagnostic is commonly used in ppls (where it is referred to as $\hat{R}$) to ascertain if the sampler has correctly sampled from the posterior. Stan’s documentation states that an $\hat{R}$ value below 1.05 passes their internal diagnostic check.

### C Software Packages

Table 3 outlines software packages that implement Bayesian inference algorithms that have been used when modelling AMR. Software packages for performing Bayesian analysis using MCMC methods with random walk proposals include WinBUGS [90], BayesianTools [91], BEAST [92] and DRAM [63]. As explained in Section IV-A1, random walk proposals can suffer from the problem of *curse of dimensionality*.

HMC and NUTS, outlined in Sections IV-A5 and IV-A7, respectively, overcome this problem by using gradient information to generate efficient proposals. Stan [70] is a ppl that performs Bayesian inference on user defined statistical models with NUTS. Stan has been extensively developed since its creation and has variants written in the programming languages C++, Python and R. Undertaking calibration using Stan requires statistical models to be deterministic [37], [41], [45], [51].

The particle filter outlined in Section IV-E1 can be described in the software package BayesianTools and the R package: pomp [93] which has the functionality of performing Bayesian inference with p-MCMC in Section IV-E2.

When the likelihood evaluation is intractable the ABC methods outlined in Section IV-C can be used. The EasyABC software package, created in R, implements an SMC sampler that uses ABC to estimate the likelihood.

## VI Popular Methods For Modeling Amr

The purpose of this section is to present well-known approaches for incorporating Bayesian principles in modeling AMR. These approaches encompass mixture models, hierarchical Bayesian models, as well as deterministic and stochastic epidemiological models. Examples of AMR applications are specified, where available. In Section VIII, we explore potential advancements and developments for these models in the future.

### A Mixture Models

Is it often the case that complex data has visible underlying sub-populations or clusters which cannot be adequately described by a one-dimensional Gaussian distribution. A Gaussian Mixture Model (GMM) is a probabilistic model that assigns and defines the probability of each datum coming from a specific cluster. A GMM can be defined as:

(47) $$p(\theta )=\sum_{i=1}^{K}w_{i}\cdot N\left(\theta ;\mu_{i},\sum_{i}\right),$$

where *p*(***θ***) is the probability density function of the GMM at ***θ***, *K* is the number of Gaussian components and *w*_*i*_ are the mixture weights, which are probabilities that sum to 1. GMMs in a Bayesian framework require prior distributions to be placed over ***θ***. The mixture weights and parameters will therefore be random variables and in this context, (47) can be defined as

(48) $$p(\theta |y)=\sum_{i=1}^{K}{\hat{w}}_{i}\cdot N\left({\hat{\mu }}_{i},{\hat{\text{Σ}}}_{i}\right),$$

where **y** is the data and $\hat{w},\hat{\mu }$ and $\hat{\sum }$ are estimated using expectation-maximization (EM). Mixture models have been successfully applied to in vitro studies [38], [51], [52] (see Section VII-C for more information) and phylogenetic trees [14], [15] (see Section VII-D for more information) when modelling AMR.

### B Hierarchical Bayesian Model

Hierarchical or multi-level Bayesian modeling is a powerful approach that involves breaking down complex statistical models into a series of interconnected sub-models arranged in a hierarchical structure. This framework allows for the combination of information from various sub-models, introducing additional uncertainty that can be beneficial in the inference process. The interdependence of priors among parameters contributes to this added uncertainty. Multiple examples of hierarchical models and AMR can be found in [50], [53], and [57].

In (2), the likelihood function, *p*(**y**|***θ***), is a function of ***θ***. However, in hierarchical Bayesian models the likelihood is a function of ***θ*** and hyper-parameters, *ϕ*, such that it is defined to be

(49) p(y∣θ,ϕ),

with

(50) p(θ,ϕ),

as its prior. Using the definition of conditional probability, (50) can be defined to be

(51) p(θ,ϕ)=p(θ∣ϕ)p(ϕ).

Using Bayes theorem, the posterior distribution can then be defined to be

(52) p(θ,ϕ∣y)∝p(y∣θ,ϕ)p(θ,ϕ),

(53) ∝p(y∣θ,ϕ)p(θ∣ϕ)p(ϕ).

As the dependence of parameters are modelled conditionally, the Gibbs sampler, outlined in section IV-A2, is a suitable MCMC algorithm to perform inference.

Hierarchical models may account for variability at different levels, such as variations between different patient populations, geographic regions, or strains of microorganisms. Data from hospital wards or separate ICUs within hospitals are often highly interdependent. Therefore, complex mathematical transmission models can be split into sub-models. For example, the colonisation status of infected patients in one ward can affect patients in another [50], [53], [57]. Each hospital ward or ICU has different parameters which are intrinsically linked.

### C Epidemiological Antimicrobial Resistance

Two common approaches for representing and simulating AMR are Compartmental Models (CM) and population-based Agent-Based Models (ABMs).

CMs divide the population into distinct compartments, each representing a different health state. The transitions between these compartments are governed by a set of differential equations (see Section IV-D1).

Incorporating stochasticity within the SIS model (see Section IV-D1) can better reflect real life scenarios [21], [23], [28], [30], [36], [39], [45], [47], [48], [59]. Stochastic fluctuations to the disease dynamics can be added to the discrete ODEs in (22) by adding a noise term, *ϵ*_*x*_, for each time-varying parameter. The noise mimics random interactions between individuals within the population. The stochastic SIS model is then defined as

(54) $$S_{t+1}=S_{t}-\frac{\beta I_{t}S_{t}}{P_{op}}-\gamma I_{t}-\epsilon_{\beta }+\epsilon_{\gamma }\text{Δ}t,$$

(55) $$I_{t+1}=I_{t}+\frac{\beta I_{t}S_{t}}{P_{op}}+\gamma I_{t}+\epsilon_{\beta }-\epsilon_{\gamma }\text{Δ}t,,$$

where $\epsilon_{\beta }\sim N\left(0,\sqrt{\beta }/P_{op}\right)$ and $\epsilon_{\gamma }\sim N\left(0,\sqrt{\gamma }/P_{op}\right)$.

References [21], [24], and [47] stress the importance of stochastic models when working with data from small populations as stochastic effects can become highly important. Reference [47] collects samples of multidrug-resistant *A. baumannii* from 15 patients in a hospital ward and report a Stochastic Differential Equation (SDE) is necessary, over an ODE, to obtain reliable estimates. They do this by adding a continuous-time Markov process to the ODE which results in an SDE. In a study of cefixime resistance in *N. gonorrhoea* [59], it was shown that another advantage of using a stochastic model over the deterministic equivalent is that the small number of cases in the early and late stages of the outbreak would not be captured by their deterministic model.

Individual ABMs are another method for simulating interactions with individuals within a population based on a set of rules [30], [36], [45]. ABMs are useful for modeling individual-level behaviors, such as antibiotic usage and transmission dynamics. They can capture heterogeneity in the population and simulate the emergence and spread of AMR at the individual level. ABMs relax the homogeneity assumption, which underpins traditional CMs. Although CMs do simulate interactions with individuals in a population, they don’t represent them explicitly like ABMs. This has been argued as a major advantage when making inferences about nosocomial transmission of MRSA [30]. ABMs can easily express the heterogeneity of patient and health care worker contacts within different wards of a hospital. The flexibility of individual ABMs is also an advantage when compared to ODE or SDE based models. For example, it is shown in [36] that the colonisation rate of extended spectrum beta-lactamase producing *E. coli* and *K. pneumoniae* is affected by factors including time-of year, coinhabitant information and individual related information such as gender, income and age, which can easily be modelled using ABMs.

#### 1) Methods To Prevent and Control AMR

**Figure 7:**
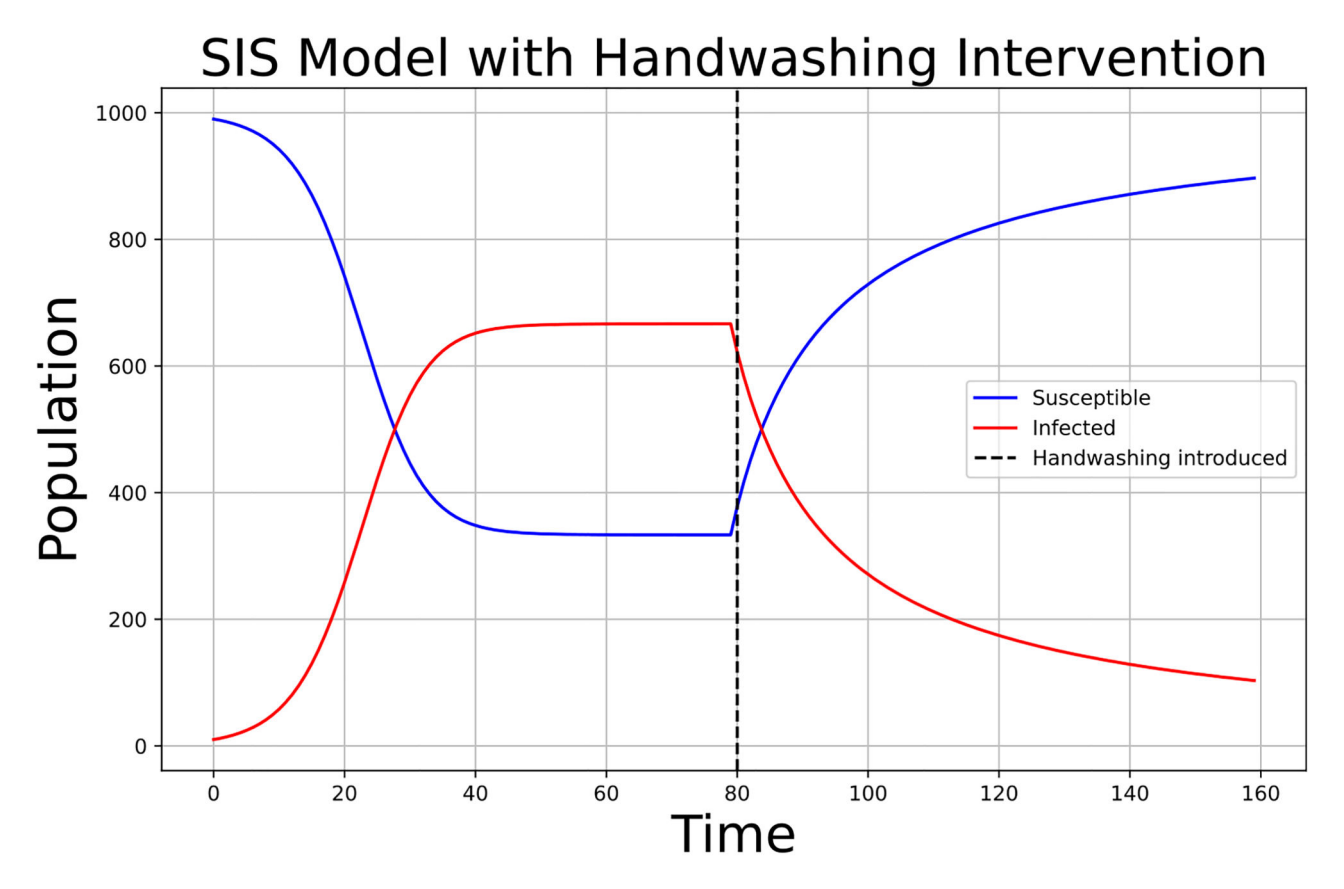
Simulation of the discrete time approximation of the ODEs in (22) and (23). Hand hygiene by health-care workers is introduced at *t* = 80 (black dashed vertical line) with *β* = 0.3 and *β* = 0.1 pre and post hand hygiene intervention, respectively.

In epidemiological studies, identifying and implementing effective measures to prevent and control AMR within specific populations is a critical area of focus. Healthcare settings, in particular, have garnered significant attention due to the heightened risk of patients acquiring infections caused by drug-resistant bacteria [23], [24], [25], [29], [30], [54], [57]. Assessing whether a patient is colonised with resistant bacteria upon arrival at a healthcare facility is crucial to prevent the potential spread to other inpatients. Methicillin resistant *S. aureus*, in particular, is a focus of concern given the severity of infections it can cause [23], [24], [25], [29], [30]. The use of antimicrobials to manage MRSA rates may lead to unintended collateral AMR, with manifestations occurring over months or years, extending beyond the typical follow-up period of clinical studies. Simulation studies, particularly those employing Bayesian calibration, can effectively model long-term AMR outcomes. For instance, a simulation study revealed that the long-term consequence of a “universal” mupirocin usage policy was the development of mupirocin resistance [23]. Interventions, such as incorporating improved hand hygiene by health-care workers [24], [29], [57], decolonisation measures e.g., enhanced cleaning of medical equipment and the environment [29], [54], [57] and limiting patient exposures and contact precautions via isolation rooms for colonised patients [25], [54], [57] could be beneficial. Figure 7 shows the impact of implementing improved hand-hygiene measures when simulating the discrete time approximation of the ODEs in (22) and (23). The change of the transmissibility parameter *β* from 0.3 to 0.1, when the measures are introduced at *t* = 80, has an effect on the number of individuals in the infected compartment. However, employing blanket package interventions of isolation, testing and decolonisation treatments to all patients can consume valuable resources [25]. Therefore, an approach targeted to high risk patients could be more desirable [30]. Employing individual, patient-level compartmental transmission models that depict Intensive Care Units (ICUs) or hospital wards allows for the monitoring of each patient’s status while intervention and control strategies are enacted [23], [24], [25], [30]. The implementation of control strategies in real-world scenarios during data collection [23], [25], [29], [54], [57] or through sensitivity analysis [24], [30], such as adjusting parameters to explore various scenarios (e.g., modifying a hand hygiene parameter to assess the impact of healthcare workers’ adherence to handwashing), enables the identification of changes in bacteria transmission dynamics [24].

Enhancing adherence to hand hygiene is identified as the most effective measure in preventing the transmission of MRSA, as evidenced by Bayesian estimates of the transmission count per uncolonized patient, referred to as the attack rate in [24]. An MCMC algorithm found that including isolation and decolonisation measures reduced the transmission dynamics of MRSA by 64% in [25].

## VII Antimicrobial Resistance

This section describes how studies have examined AMR. As explained in Section I, an effective AMS is multi-faceted and aims to integrate various initiatives. Among these initiatives are programs addressing multi-drug resistance, economic implications, and modeling AMR in vitro as well as within specific populations. The subsequent paragraphs provide an overview of these programs.

### A Drug/Multi-Drug Resistance

The global trend of multi-drug resistance is a cause for concern. In 2019, an estimated 1.27 million deaths worldwide were attributed to drug and multi-drug resistance, with AMR-resistant infections contributing to 4.95 million deaths [96]. A significant contributor to these fatalities, numbering approximately 50,000 to 100,000, is multi-drug-resistant *M. tuberculosis*, a form of *M. tuberculosis* resistant to isoniazid and rifampin, the two most potent anti *M. tuberculosis* medications. Bayesian modeling and the estimation of anticipated cases, incidence, and mortality related to multi-drug-resistant *M. tuberculosis* constitute an active and ongoing area of research [13], [14], [16], [17], [20]. Bayesian analysis of other pathogens resistant to multiple drugs include: *Salmonella* spp [51], [52]; *E. coli* [34], [35]; *N. gonorrhoea* [41] and *A. baumannii* [47].

### B Economic Cost

As stated in [97], the rise of AMR is associated with an annual increase of nine billion euros and 20 billion dollars in healthcare costs in Europe and the United States, respectively. With diminishing effectiveness of antimicrobials against diseases, these costs are expected to increase. Two noteworthy studies, focusing on the epidemiological and public health impact, as well as the cost-effectiveness of implementing vaccine programs for *N. gonorrhoea* [40] and *M. tuberculosis* [20], exist. Both studies employ integrated transmission-dynamic compartmental health-economic models to project cost savings based on vaccine rollout. According to [20], *M. tuberculosis* vaccination is poised to significantly reduce future case burdens, while [40] advocates for a targeted vaccination approach for better economic outcomes. Utilizing Bayesian methods for calibration is well-suited for this analysis, given the inherent uncertainty in model parameters and cost inputs, as highlighted in [20].

### C Modelling of in vitro Phenomena

Bayesian calibration of in vitro studies has been an active area of research [18], [31], [35], [38], [41], [51], [52], [58], [98]. The Minimal Inhibitory Concentration (MIC) establishes the susceptibility or resistance of antibiotics when tested against antimicrobials in vitro. It signifies the minimum antibiotic concentration at which detectable bacterial growth is observed. A lower MIC value suggests that a smaller drug concentration is needed to inhibit bacterial growth, making it more desirable. MIC serves as a benchmark for comparing antimicrobial agents across various studies. For example, *N. gonorrhoea* was tested against ciprofloxacin, cefixime and azithromycin in [41] and ciprofloxacin, penicillin, and tetracycline in [58]. The same classes of antibiotics can also be applied to different bacteria for example, tetracycline tested against *E. coli* [35] and *N. gonorrhoea* [58] and amoxicillin tested against *E. coli* [35] and *Salmonella* spp [51].

MIC experiments can be resource-intensive and expensive due to the necessary equipment. The potential for human error in recording MIC readings is also a problem. Recent work, named AIgarMIC [99], has attempted use ML to automate the readings of MIC experiments. The observation of bacterial growth frequently leads to censored data, where observations fall within fixed time intervals between dilution experiments. This limitation arises because the exact time of growth occurs within the interval from the last dilution inhibiting growth to the first dilution where no growth is visible. Approaches to address censored data within a Bayesian framework are discussed in [31], [38], [51], [52], and [58].

Among the in vitro studies discussed previously, two prevalent methods for modeling MIC are noteworthy. Examples include ODE based models, such as those describing how single or multiple drug resistance mutations influence MIC distribution [41]. These models also explore noninherited resistance levels in bacteria when exposed to antibiotics [35], as well as the emergence of antibiotic-resistant subpopulations in *M. tuberculosis* cells treated with isoniazid [18]. The second method involves hierarchical mixture models, exemplified by their ability to represent subpopulations of antibiotic resistance within an overall population of isolates [38], [51], [52].

### D Specialised Areas of AMR

This review revealed two specialized areas of research related to AMR, briefly discussed here. Firstly, several studies incorporated the analysis of molecular sequences associated with drug resistance using phylogenetic trees [14], [15], [22], [32], [33], [42], [44], [94]. As seen in Table 3, the software Bayesian Evolutionary Analysis Sampling Trees (BEAST) [92] is widely used for conducting Bayesian analysis on the evolution of drug-resistant genomes. Additionally, the software package Tracer [100] is commonly employed to visualize the MCMC traceplots generated by the BEAST software. The second specialized area of research pertains to popPK models. These models aim to depict relationships between patient characteristics and drug exposure, with a focus on inferring the susceptibility of the drug to the microorganism and the exposure to the drug [101], [102].

## VIII Recent Advancements and Scope for Future Work

This section outlines potential avenues for future work with regards the applicability of the latest advancements and applications in Bayesian modeling of AMR. As is with healthcare in general, AMR is a field that is dynamic and continuously evolving.

### A Combination Approach to AMR

A

This review has outlined studies that have attempted to address the measures outlined in Section I which constitute an effective AMS. According to [8], the challenges arise when combining these measures in a coherent framework which is interpretable by clinicians and healthcare workers and adheres to the legal requirements needed to utilise in the healthcare system. Examples of how future research could integrate multiple areas discussed in this review include:

- Extend the use of hierarchical Bayesian models to integrate data from multiple scales e.g. combining MIC laboratory testing of antimicrobials with broader surveillance of AMR in target populations to better understand the transmission dynamics of AMR.
- Integrating epidemiological and genomic data to identify genetic markers associated with resistance and track the evolution of resistance genes could follow a similar approach to that described by [103] for HIV. In their study, they calibrate time series prevalence data and dated phylogeny reconstructed from sequences to infer the reproduction number using p-MCMC. Given the potential computational expense of this approach, especially with long time-series data, employing SMC^2^ could be advantageous.
- Economic and policy models could be combined using Bayesian methods to quantify the economic impact of AMR and evaluate the cost-effectiveness of different intervention strategies.

### B Sampling Algorithms

The progression from the MHRW proposal in MCMC to gradient-based methods is clearly outlined in this review. It is evident from Table 2 that the majority of studies identified in the search criteria are random walk based algorithms. In the following paragraphs, we intend to describe recent advancements in p-MCMC, SMC^2^, and SMC samplers.

As detailed in Section IV-E2, the standard proposal utilized in p-MCMC and SMC samplers is MHRW, which can face challenges associated with the *curse of dimensionality*. Recent advancements have explored the use of MALA, HMC, and NUTS as proposals within p-MCMC [104], [105]. Similarly, recent advancements with gradient-based proposals within SMC samplers can be found in [106] and [107]. One notable challenge in p-MCMC and MCMC methods lies in their inherent sequential nature, limiting efficient parallelization. Multichain MCMC is one method that can efficiently explore *π*(***θ***) with [108] presenting a framework for running on GPUs. To overcome this constraint, a potential solution involves replacing MCMC with an SMC sampler and p-MCMC with SMC^2^. Recent studies have shown how to implemented these algorithms on high-end computing facilities [36], [109]. A specific example can be found in [36] where they implement SMC^2^ on GPU nodes when calibrating transmission models pertinent to the spread of *E. coli*.

### C Differentiable Agent Based Models

ABMs and equation-based CMs represent two frameworks employed for modeling the spread of communicable diseases within populations. ABMs diverge from the homogeneity assumption inherent in traditional CMs by simulating heterogeneous agents with predefined behaviors. The challenges of scalability when simulating millions of individuals and the non-differentiable nature of ABMs have been perceived as hindrances to widespread use. Recent efforts, such as those highlighted in [110], have focused on developing differentiable ABMs. A notable example is GradABM [111], which introduces a scalable, differentiable ABM capable of simulating populations in the millions within a few seconds and accommodating heterogeneous data sources related to COVID-19. A promising avenue for future research would involve calibration of differentiable ABMs to AMR data using gradient-based MCMC and SMC samplers.

### D Specialised Areas of AMR

In Section VII-D, two specialized areas of AMR are discussed: phylogenetic trees and popPK models. The software package BEAST is a prominent choice for Bayesian analysis of molecular sequences using MCMC. A recent review has outlined practical guidelines for Bayesian phylogenetic inference using MCMC [112]. As the size and complexity of data and models increase, so does the runtime required to obtain meaningful results. Recent advancements, as discussed in [113], introduce an SMC sampler specifically designed for decision trees, and this implementation is compatible with shared memory architectures. The study demonstrates that SMC samplers are as accurate as MCMC but faster, offering potential benefits in this context. Concerning popPK models, the use of gradient based MCMC algorithms, such as HMC, could be a beneficial alternative to likelihood statistical methods, as outlined in the review article [114]. A software package named Torsten [115] utilizes functions derived from the ppl Stan to tackle popPK related problems. While both areas necessitate further exploration, they represent intriguing directions for future research.

## IX Conclusion

This review article explores the application of Bayesian methods in understanding AMR. It examines various studies that employ Bayesian statistical techniques to model and predict the dynamics of AMR. The paper outlines the evolution of sampling algorithms, detailing the shift from traditional random walk methods to more advanced gradient-based approaches. These advancements have improved the accuracy and efficiency of AMR modeling, offering deeper insights into the spread and control of resistance.

It is evident from this review that a combination approach to antimicrobial AMR stewardship should be employed to optimize the use of antimicrobial agents, reduce the spread of resistance, and improve patient outcomes. As computational resources increases, the ability to analyse AMR related data in real time will become hugely important. One such example is phylogenetic analysis and the ability to detect mutations that are resistant to antibiotics.

As AMR continues to pose a global health threat, the application of Bayesian techniques will be crucial in developing effective strategies for monitoring and mitigating resistance. Future research should focus on further refining these methods and exploring their integration with other computational tools to enhance our understanding and management of AMR.

## Figures and Tables

**Table 1:** A list of pathogens and corresponding studies that were included in this review. The process of selecting studies is outlined in Section I-A.

Pathogen	Studies
*Mycobacterium tuberculosis*	[13]–[22]
Methicillin-resistant *Staphylococcus aureus and methicillin-sensitive Staphylococcus aureus*	[23]–[33]
*Escherichia coli*	[34]–[38]
*Neisseria gonorrhoea*	[39]–[44]
*Klebsiella pneumoniae*	[36], [37], [45]
*Acinetobacter baumannii*	[46], [47]
*Streptococcus pneumoniae*	[48], [49]
*Pseudomonas aeruginosa*	[50]
*Salmonella* spp	[51], [52]
*Enterococcus* spp	[53], [54]

**Table 2:** A list of Bayesian algorithms and corresponding studies that were selected in the review article. The process of selecting studies is outlined in Section I-A.

Sampling Algorithm	Proposal	Studies
ABC	-	[13], [20], [21], [30], [48]
MCMC	MH	[14]–[18], [27], [34], [35], [40], [46], [47], [54], [57], [58]
Adaptive MCMC	MH	[18], [19], [57]
MCMC	Gibbs	[26], [28], [38], [50], [52]–[54]
MCMC	Langevin	[38]
MCMC	NUTS	[31], [37], [41], [44], [45], [51]
SMC Sampler	ABC proposal	[43]
Particle-MCMC	MH	[39], [59]
SMC^2^	MH	[36]

**Table 3:** Software packages that have been used to perform Bayesian inference on models relating to AMR.

Software Package	Ref.	Description	Studies
Stan	[70]	MCMC with NUTS	[31], [37], [41], [44], [45], [51]
WinBUGS	[90]	MCMC with Gibbs	[14], [15], [58]
R package: pomp	[93]	particle filters, p-MCMC, ABC	[39], [59]
BayesianTools	[91]	MCMC with RW, particle filters	[16], [57]
BEAST	[92]	MCMC with RW	[22], [32], [42], [94]
DRAM	[63]	Adaptive MCMC	[18]
Easy ABC	[95]	SMC sampler with ABC	[43]
